# Reduction of Matrix Metallopeptidase 13 and Promotion of Chondrogenesis by Zeel T in Primary Human Osteoarthritic Chondrocytes

**DOI:** 10.3389/fphar.2021.635034

**Published:** 2021-05-11

**Authors:** Christelle Sanchez, Kathrin Hemmer, Natascha Krömmelbein, Bernd Seilheimer, Jean-Emile Dubuc, Christophe Antoine, Yves Henrotin

**Affiliations:** ^1^ MusculoSKeletal Innovative Research Lab, University of Liège, Center for Interdisciplinary Research on Medicines, Liège, Belgium; ^2^ Heel GmbH, Baden-Baden, Germany; ^3^ Division of Orthopedics and Musculoskeletal Trauma, Cliniques Universitaires de St Luc, Brussels, Belgium; ^4^ Artialis SA, Liège, Belgium; ^5^ Physical Therapy and Rehabilitation Department, Princess Paola Hospital, Vivalia, Marche-en-Famenne, Belgium

**Keywords:** osteoarthritis, cartilage, chondrogenesis, zeel T, MMP13, multitarget

## Abstract

**Objectives:** Zeel T (Ze14) is a multicomponent medicinal product. Initial preclinical data suggested a preventive effect on cartilage degradation. Clinical observational studies demonstrated that Ze14 reduced symptoms of osteoarthritis (OA), including stiffness and pain. This study aimed to explore these effects further to better understand the mode of action of Ze14 on human OA chondrocytes *in vitro*.

**Methods:** Primary chondrocytes were obtained from the knees of 19 OA patients and cultured either as monolayers or in alginate beads. The cultures were treated with 20% or 10% (v/v) Ze14 or placebo. For RNA-seq, reads were generated with Illumina NextSeq5000 sequencer and aligned to the human reference genome (UCSC hg19). Differential expression analysis between Ze14 and placebo was performed in R using the DESeq2 package. Protein quantification by ELISA was performed on selected genes from the culture medium and/or the cellular fractions of primary human OA chondrocyte cultures.

**Results:** In monolayer cultures, Ze14 20% (v/v) significantly modified the expression of 13 genes in OA chondrocytes by at least 10% with an adjusted *p*-value < 0.05: EGR1, FOS, NR4A1, DUSP1, ZFP36, ZFP36L1, NFKBIZ, and CCN1 were upregulated and ATF7IP, TXNIP, DEPP1, CLEC3A, and MMP13 were downregulated after 24 h Ze14 treatment. Ze14 significantly increased (mean 2.3-fold after 24 h, *p* = 0.0444 and 72 h, *p* = 0.0239) the CCN1 protein production in human OA chondrocytes. After 72 h, Ze14 significantly increased type II collagen pro-peptide production by mean 27% (*p* = 0.0147). For both time points CCN1 production by OA chondrocytes was correlated with aggrecan (r = 0.66, *p* = 0.0004) and type II collagen pro-peptide (r = 0.64, *p* = 0.0008) production. In alginate beads cultures, pro-MMP-13 was decreased by Ze14 from day 7–14 (from −16 to −25%, p < 0.05) and from day 17–21 (−22%, *p* = 0.0331) in comparison to controls.

**Conclusion:** Ze14 significantly modified the expression of DUSP1, DEPP1, ZFP36/ZFP36L1, and CLEC3A, which may reduce MMP13 expression and activation. Protein analysis confirmed that Ze14 significantly reduced the production of pro-MMP-13. As MMP-13 is involved in type II collagen degradation, Ze14 may limit cartilage degradation. Ze14 also promoted extracellular matrix formation arguably through CCN1 production, a growth factor well correlated with type II collagen and aggrecan production.

**GRAPHICAL ABSTRACT:**
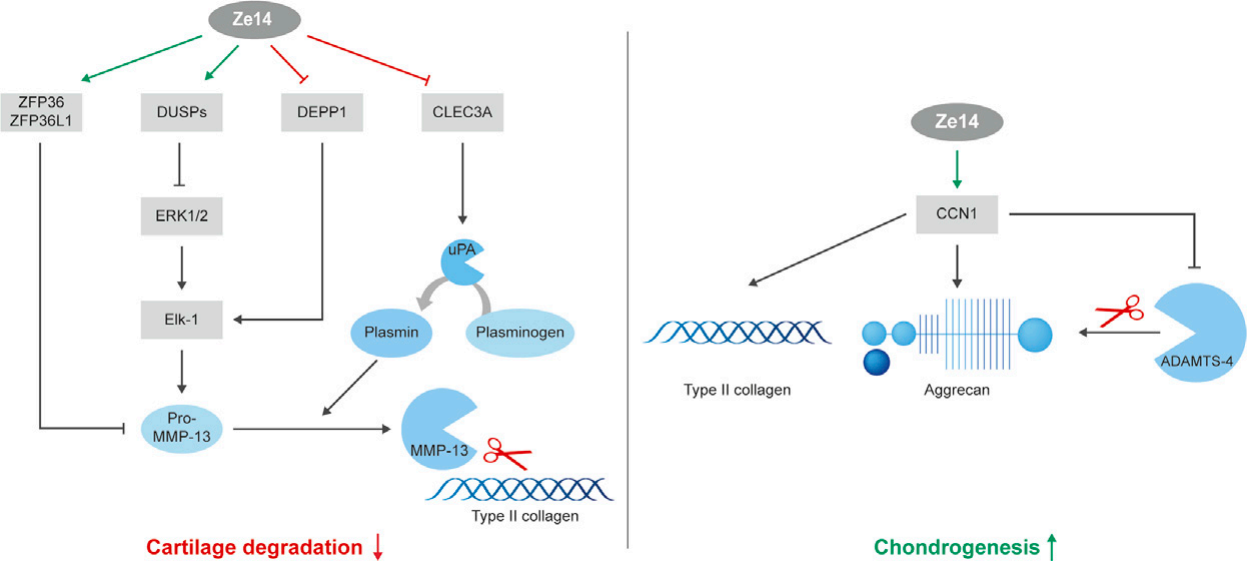


## Introduction

Osteoarthritis is the most common form of arthritis, affecting millions of people worldwide. It is a serious disorder primarily affecting weight bearing joints characterized by cell stress and extracellular matrix degradation initiated by micro- and macro-injury that activates maladaptive repair responses, including proinflammatory pathways of innate immunity. The disease manifests first as a molecular dysregulation (abnormal joint tissue metabolism) progressing to anatomical derangements (characterized by cartilage degradation, bone remodeling, osteophyte formation, joint inflammation, and loss of normal joint function) (Kraus et al., 2015). It primarily affects the elderly population. Due to an increasing number of OA patients, finding a disease-modifying OA drug, defined as “a drug that inhibits structural disease progression and also improves symptoms and/or function” (Barr and Conaghan, 2013), remains an urgent unmet need. Currently, despite the guidelines, first-line treatment of OA in daily medical practice remains with analgesics and anti-inflammatory drugs, although it is recognized that the long-term use of these drugs is linked to severe adverse effects (Bannuru et al., 2019). The recommendations published by medical and scientific societies are indeed unanimous: their use must be limited. Therefore, it is essential to find alternative treatments to manage the symptoms of patients with OA as well as to inhibit structural disease progression (Geenen et al., 2018; Rausch Osthoff et al., 2018; Bannuru et al., 2019). Due to the complexity of the disease, multitarget treatments seem to be more suitable to target the multiple pathological pathways associated with OA. Zeel T (Ze14) is a multicomponent medicinal product composed of plant and organ extracts. Clinical observational studies demonstrated that Ze14 reduced symptoms of OA, including stiffness and pain, and was generally well-tolerated with a good safety profile (Gottwald and Weiser, 2000; Lesiak et al., 2001). Initial preclinical data suggested a preventive effect of Ze14 on cartilage degradation (Stancikova, 1999); however, Ze14’s mode of action is still poorly understood. Due to its promising multitarget nature and the lack of effective treatment options for OA patients, investigating the mode of action of Ze14 in OA pathophysiology may provide important insights explaining at least some effects observed in the clinical studies with OA patients.

Recently, transcriptome analysis was used to investigate the effects of natural or chemically produced molecules on chondrocyte transcriptome *in vitro* (Gouze et al., 2006; James et al., 2007; Comblain et al., 2016; Aury-Landas et al., 2017). Technologies like Next Generation Sequencing RNA-seq generate an unbiased view of the transcriptome offering a wider dynamic range, high sensitivity, and accurate results on all genes expressed by cells (Wang et al., 2014; Liu et al., 2015; Li et al., 2016).

This exploratory study aimed to investigate the mode of action of Ze14 in OA by comparing the transcriptomic profile of human OA chondrocytes treated with Ze14 or saline as control. The transcriptomic profile of OA chondrocytes was determined from primary cell cultures treated with Ze14 at two concentrations, with or without the addition of IL-1β to activate inflammatory pathways. Human primary cells retain the morphological and functional characteristics of their tissue of origin. Thus, we chose this primary OA cell model to reflect the pathological processes of knee OA, also considering patient to patient variations. Affected genes of the most relevant disease pathways were then confirmed by protein analysis in additional chondrocyte cultures.

## Material and Methods

### Ethical Statement

Articular cartilage samples from 19 patients with knee OA were obtained at the time of total knee joint replacement (TKR) surgery. All participants have signed the informed patient consent, and the protocol was approved by the ethical committee of the Catholic University of Louvain (B403201214793 amendment n°2). All procedures followed the ethical standards of the responsible committee on human experimentation (institutional and national) and with the Helsinki Declaration of 1975, revised in 2000. The Supplementary File S1 gives an overview of the patients’ characteristics.

### Study Medication

Zeel T (Ze14) injection solution was manufactured and bottled in 2.0 mL glass ampoules by Heel GmbH, Germany, according to GMP standards. The study medication was packaged, shipped, and labeled by Heel GmbH, Germany. The full composition of Ze14 injection solution is provided in the Supplementary File 2. Each 1.1 mL ampoule of the saline control contained 0.9% sodium chloride for injection.

### Study Design

The cartilage samples from the patients were divided into two groups, one for sequencing and transcriptome generation, and another for protein quantification by ELISA. The harvested tissue was enzymatically digested and seeded in monolayer cultures or suspended in alginate beads depending on the analysis. Supplementary File 3 gives a detailed overview of the study design and explains the distribution of tissue samples for the primary cultures, number of treatment conditions including controls, time points of sample collection for analyses, and performed analyses. In short, for transcriptome analysis, chondrocytes were treated with Ze14 or saline control with or without the presence of IL-1β to induce the inflammatory state. Two concentrations of Ze14, 10% (v/v) and 20% (v/v) were used. For protein analysis, chondrocytes were seeded as monolayers or suspended in alginate beads to investigate chondrocyte hypertrophy. Both cultures were treated with either 20% (v/v) Ze14 or saline control in medium.

### Sample Collection

Full-depth articular cartilage was excised and immersed in Dulbecco’s Modified Eagle Medium (DMEM) (with phenol red and 4.5 g/L glucose) supplemented with N-(2-hydroxyethyl)piperazine-N’-(2-ethanesulfonic acid) (HEPES) (10 mM), penicillin (100 U/mL) and streptomycin (0.1 mg/mL) (all from Lonza, Belgium). After three washes, chondrocytes were released from cartilage by sequential enzymatic digestions with 0.5 mg/mL hyaluronidase type IV S (Sigma-Aldrich, Belgium) for 30 min at 37°C, 1 mg/mL pronase E (Merck, Belgium) for 1 h at 37°C and 0.5 mg/mL clostridial collagenase IA (Sigma-Aldrich, Belgium) for 16–20 h at 37°C. The enzymatically isolated cells were then filtered through a nylon mesh (70 µm), washed three times and counted.

### Chondrocyte Monolayer Culture

Chondrocytes were dispersed in a suspension of 0.1 x 10^6^ cells/mL in DMEM supplemented with 10% fetal bovine serum (Biowest, France), 10 mM HEPES, 100 U/mL penicillin, 0.1 mg/mL streptomycin, 2 mM glutamine (Lonza, Belgium), 20 μg/mL proline and 50 μg/mL ascorbic acid (Sigma-Aldrich, Belgium) and seeded in 6-well plates at the density of 0.2 x 10^6^ cells/well. Chondrocytes were cultured in monolayer for 5–7 days until 95% confluence. Only primary cultures were used to ensure the stability of the chondrocyte phenotype. Chondrocytes were then cultured 24 h in DMEM supplemented with 1% fetal bovine serum, 10 mM HEPES, 100 U/mL penicillin, 0.1 mg/mL streptomycin, 2 mM glutamine, 20 μg/mL proline and 50 μg/mL ascorbic acid. Afterward, the culture medium was replaced by fresh culture medium containing either 20% (v/v) saline (control), 20% (v/v) Ze14 or 10% (v/v) Ze14 (lower concentration was achieved as follows: 8 parts medium: 1 part saline: 1 part Ze14; corresponding to half of the Ze14 concentration, used only for the transcriptomic study), with or without the addition of human IL-1β 10^–11^ M (Roche, Belgium) (Comblain et al., 2016). Cells were incubated for 24 h (transcriptome analysis only) or 72 h (transcriptome and protein analysis). Each culture condition was carried out in triplicates.

For transcriptome analysis, cells were scrapped after 24 h of incubation, three wells were pooled, and ribonucleic acid (RNA) extraction was performed using an RNeasy mini kit according to the instructions of the manufacturer (Qiagen, Netherlands). Cell lysates were stored frozen at −80°C until RNA extraction.

For LDH release assay, conditioned culture media was collected after 24 h of incubation and assayed immediately. Cells were scraped and homogenized in 500 µL of Tris-HCl buffer by ultrasonic dissociation for 20 s at 4°C, to measure total LDH content. Remaining conditioned culture media was stored at −20°C until further analysis.

For protein analyses, conditioned culture media was collected after 24 h and 72 h of incubation and was then stored at −20°C until further analysis. Three wells were used per condition. Cells were trypsinized and homogenized in 500 µL of Tris-HCl buffer by ultrasonic dissociation for 20 s at 4°C to measure total DNA content after 24 h and 72 h of incubation.

### Chondrocyte Alginate Beads Culture

Chondrocytes were suspended in alginate beads (as described (Sanchez et al., 2002)) and cultured for 28 days in Dulbecco’s modified Eagle’s medium supplemented with 10% FBS, 10 mM HEPES, penicillin (100 U/mL) and streptomycin (100 μg/mL), 200 μg/mL glutamine, 50 μg/mL ascorbic acid, and 2 mM proline to induce hypertrophy (Pesesse et al., 2013). Alginate beads containing chondrocytes were placed in 24-well plates, nine beads per well. Six wells were used per time point and treatment: three wells per condition were used for the analyses of the alkaline phosphatase activity and the protein pro-MMP-13 production, and three other wells for RNA extraction and type X collagen gene expression.

Ze14 or saline control was added at 20% (v/v) in the culture medium with every culture medium replacement (from day 0 to day 24). The culture medium was changed twice a week.

At each time point (Day 3-7-14-17-21-24-28), cell culture was stopped, and supernatant was collected and stored at −20°C for further analysis. Additionally, supernatant was collected on day 10. The beads of each well were rinsed in NaCl 0.9% and then dissolved in 1 mL of 0.1 M citrate for 10 min. The resulting suspension was centrifuged at 1200 rpm for 10 min. With this method, 2 fractions were collected: the supernatant containing macromolecules that originated from further-removed matrix (FRM) and pellet-containing cells with their associated matrix (CM). These two fractions CM and FRM were kept separately at −20°C until analysis. The supernatant from the FRM was not used for the analyses. CM of 3 wells was homogenized in 500 µL of Tris-HCl buffer by ultrasonic dissociation for 20 s at 4°C to measure total DNA content and alkaline phosphatase activity. The CM of the remaining 3 wells were homogenized in Lysis buffer for ribonucleic acid (RNA) extraction using RNeasy mini kit according to the instructions of the manufacturer (Qiagen, Netherlands). Cell lysates were stored frozen at −80°C until RNA extraction.

### LDH Viability Test

Cell viability was estimated by quantifying the release of LDH into the culture supernatant as previously described (Mathy-Hartert et al., 2009). In brief, a sample of the supernatant or dilutions of standard solution (LDH from rabbit muscle, from Roche, Belgium) was mixed with Tris buffer (10 mM Tris-HCl (pH 8.5), 0.1% bovine serum albumin) containing 800 mM lactate. Then, colorimetric reagent, 1.6 mg/mL iodonitrotetrazolium chloride (Sigma-Aldrich, Belgium), 4 mg/mL nicotinamide adenine dinucleotide (Roche Diagnostics, Belgium), and 0.4 mg/mL phenazine methosulfate (Sigma-Aldrich, Belgium), was added, and the solution was discoloured red after 10 min of incubation at room temperature. The percentage of cell death was obtained by comparing the LDH release into the supernatant to the total LDH concentration (cell and supernatant).

### DNA Quantification

DNA content of all cell cultures was measured according to a fluorometric method (Labarca and Paigen, 1980). In brief, 200 µL of 2 μg/mL Hoechst dye solution (Sigma Aldrich, Belgium)−in a buffer containing 50 mM PO_4_ and 2 M NaCl− was added to 50 µL of sample. After 30 min incubation time in the dark, 356 nm excitation/458 nm emission was read in a spectrophotometer. Placental DNA (Sigma Aldrich, Belgium) was used for the standard curve.

### RNA Extraction

Total RNA was extracted using an RNeasy mini kit (Qiagen, Belgium) according to the instructions of the manufacturer. The yield of the extracted RNA was determined spectrophotometrically by measuring the optical density at 260 nm. For transcriptome analysis, the purity and quality of extracted RNA was further evaluated using an RNA Nano 6000 Bioanalyzer Agilent (Santa Clara, United States) according to the manufacturer’s instructions. High-quality RNA with RNA quality indicator scores (RIN) of >8 were used.

### RNA-Seq and Differential Gene Expression Analysis

One µg of RNA from each culture condition was used for this analysis. Libraries were prepared with the Illumina Truseq stranded mRNA sample prep kit according to the manufacturer’s instructions. Based on poly(A) selection of mRNAs, the coding strand information was kept. Sixty (60) libraries were generated. Poly(A) plus RNA was enriched using oligo (dT) beads followed by fragmentation and reverse transcription. Afterward, the 5′ and 3′ ends of cDNA fragments were prepared to ensure efficient ligation of “Y” adapters containing a unique barcode and primer binding sites. Finally, ligated cDNAs were PCR-amplified to be ready for cluster generation and sequencing.

Sequencing was performed on Illumina NextSeq5000, Single-Read 75 bp read length, high output mode 2017–2018 (Maximum Reads per Run: 400 million clusters), 60 libraries per run, and using 3 runs per library, generating around 20 M reads per sample (Chaitankar et al., 2016). Denaturated NGS library fragments were flowed across a flow cell and hybridized on a lawn of complementary Illumina adapter oligos. Complementary fragments were extended, amplified via bridge amplification PCR and denaturated, resulting in clusters of identical single-stranded library fragments. Fragments were primed and sequenced utilizing reversible terminator nucleotides. Base pairs were identified after laser excitation and fluorescence detection.

Raw data was demultiplexed into individual libraries. After filtering out reads mapping to rRNA, tRNA, mitochondrial RNA, and other contaminants, (e.g. adapters, etc.) using bowtie2, reads were aligned onto the human reference genome (UCSC hg19 annotation) and quantified with Star to give the Counts file. Quality control of sequencing reads was assessed with FASTQC and quality control after mapping with Picard tools. Compilation of tool metrics was performed with MultiQC.

Differential expression analysis was made in R (version 3.4.3 (2017–11–30), https://www.R-project.org/) using the DESeq2 package (1.18.1) and R code design = ∼ Patient + Treatment. Analysis was performed with treatment as contrast:

• Saline control vs Ze14 20% (v/v)
• Saline control vs Ze14 10% (v/v)
• Saline control vs Ze14 20% (v/v), both with IL-1β
• Saline control vs Ze14 10% (v/v), both with IL-1β

Alternatively, the interaction “Patient:Treatment” was added to the R code design to evaluate if the treatment effect varied from patient to patient. False Discovery Rate of 0.05 was used to assess the statistical significance.

### Alkaline Phosphatase Activity

The enzymatic activity of alkaline phosphatase normalized to the DNA content of the respective well was analyzed in the alginate beads cultures according to the previously described method (Sanchez et al., 2005). In brief, 50 µL of cell extract were incubated with 100 µL of p-nitrophenylphosphate (liquid p-NPP, ready to use, Sigma Aldrich, Belgium). In the presence of ALP, p-NPP is transformed to p-nitrophenol and inorganic phosphate. p-nitrophenol absorbance is measured at 405 nm, after 10 min of incubation at 37°C. A standard preparation of p-nitrophenol was used for calibration. Results were expressed in nmoles of p-nitrophenol released per min and per µg of DNA.

### Quantitative Real-Time RT-PCR Gene Expression

Reverse transcription was executed by using sensiscript kit according to the instructions of the manufacturer (Qiagen, Belgium), and cDNA was kept at −20°C. Polymerase chain reaction was performed by using the Rotor Gene (Qiagen, Belgium)—SYBR premix Ex Taq (Takara, Belgium). The PCR template source was either first-strand cDNA (10 ng) or purified DNA standard. The PCR program comprised an initial denaturation step at 95°C for 10 s followed by 40 cycles of denaturation at 95°C for 5 s and then an annealing/extension step at 60°C for 25 s. Followed by an ending melting step from 65°C to 96°C with a 1°C increase each second. The following primer sequences were used to amplify the desired cDNA: Hypoxanthine-guanine phosphoribosyltransferase forward 5′-TGT​AAT​GAC​CAG​TCA​ACA​GGG-3′ and reverse 5′-TGC​CTG​ACC​AAG​GAA​AGC-3′ and COL10A1 forward 5′-GGGAGTGCCATCATCG-3′ and reverse 5′-AGGGTGGGGTAGAGTT-3′. HPRT was used as an internal standard and the ratio of genes to HPRT was calculated. After HPRT normalization for COL10A1, relative expression was calculated.

### ELISA for CCN1, Pro-MMP-13, Aggrecan and Type II Collagen Pro-Peptide

Protein amount of aggrecan was assessed from the supernatant and the cell pellet while the protein amount of type II collagen pro-peptide, CCN1, and pro-MMP-13 was only measured from the supernatant, by specific enzyme amplified sensitivity immunoassays (Aggrecan: Diasource, Belgium PG EASIA KAP1461, batch 1902–2260; CCN1: CYR61 R&Dsystems UK DuoSet DY4055, batch P161032; collagenase 3 precursor -pro-MMP-13-: R&Dsystems UK DuoSet DY913, batch P196342; type II collagen pro-peptide: R&Dsystems UK DuoSet DY7589–05, batch P151876). Aggrecan, type II collagen pro-peptide, and CCN1 were assayed from chondrocytes in monolayer cultures, and pro-MMP-13 was analyzed from chondrocytes of alginate beads hypertrophic cultures. Protein content was normalized to the DNA content.

### Statistical Analysis

For the transcriptome analysis, DESeq2 Bioconductor package was used for normalization, principal component analysis, and differential gene expression. DESeq2 differential gene analysis was based on the hypothesis that most genes were not differentially expressed (Anders and Huber, 2010; Love et al., 2014). The method was based on the negative binomial distribution model. Within the DESeq2 package, and with the *estimateSizeFactorsForMatrix* function, scaling factors were calculated for each run. After dividing gene counts by each scaling factor, DESeq2 values were calculated as the total of rescaled gene counts of all runs.

The amplitude of changes is represented either in the log2 Fold Change format (classical representation from DESeq2, where “0” means “no change,” “1” means “2-fold induction” and “−1” means “2-fold decrease”) or in Fold Change (where “1” means “no change,” “2” means “2-fold induction” and “0.5” means “2-fold decrease”).

Along with the standard *p*-value, an adjusted *p*-value (padj) was calculated. The adjustment methods included the Bonferroni correction (“bonferroni”) in which the *p*-values were multiplied by the number of comparisons. Less conservative corrections were also included by Holm (“holm”) (Holm, 1979), Hochberg (“hochberg”) (Hochberg, 1988), Hommel (“hommel”) (Hommel, 1988), Benjamini and Hochberg (“BH” or its alias “fdr”) (Benjamini and Hochberg, 1995), and Benjamini and Yekutieli (“BY”) (Benjamini and Yekutieli, 2001), respectively. The “BH” (aka “fdr”) and “BY” method of Benjamini, Hochberg, and Yekutieli control the false discovery rate, the expected proportion of false discoveries among the rejected hypotheses.

For the protein analyses, results were statistically analyzed using GraphPad Prism 6.0. Before calculating the statistical difference, a Kolmogorov-Smirnov normality test and a ROUT outlier test were performed. For monolayer experiments, either a ratio paired *t*-test, or a Wilcoxon matched-pairs signed-rank test was used. For alginate beads experiments, paired one-way ANOVA was used to compare Ze14 to saline control at each time point. Furthermore, for cumulative pro-MMP-13 production, two-way ANOVA was used to evaluate the kinetic effect of the treatment. For assessing the correlation between two factors, Pearson's test was used for normal data distribution, and Spearman's test was used non-normal distribution.

## Results

### Cell Viability and RNA-Seq Database Analysis

Cell viability was higher than 98% and was not affected by IL-1β and/or Ze14 treatment, either at 10% or 20% (v/v) (Supplementary File 4). RNA-seq experiments were performed with a sequencing depth of 16.75 ± 0.52 million copies of genes per sample. IL-1β 10^**–11**^ M drastically modified the gene expression pattern of human OA chondrocytes. Principal component analysis (PCA) with regularized-logarithm transformation showed that the large variance between the samples was related to the IL-1β stimulation, explaining 86.9% of the total variance (PC1) (Supplementary File 5). The database was then split into basal and IL-1β-treated samples to continue the analysis.

### Ze14 Modulated the Expression of 13 Genes Under Basal Conditions

Under basal conditions, chondrocytes expressed genes representing a well-differentiated and mature chondrogenic cell type as confirmed by the top-count genes, with a high level of type II collagen (COL2A1–third-most counted, 6-times more than COL1A1) and aggrecan (ACAN–eighth-most counted) gene expression (Supplementary File 6).

**FIGURE 1:**
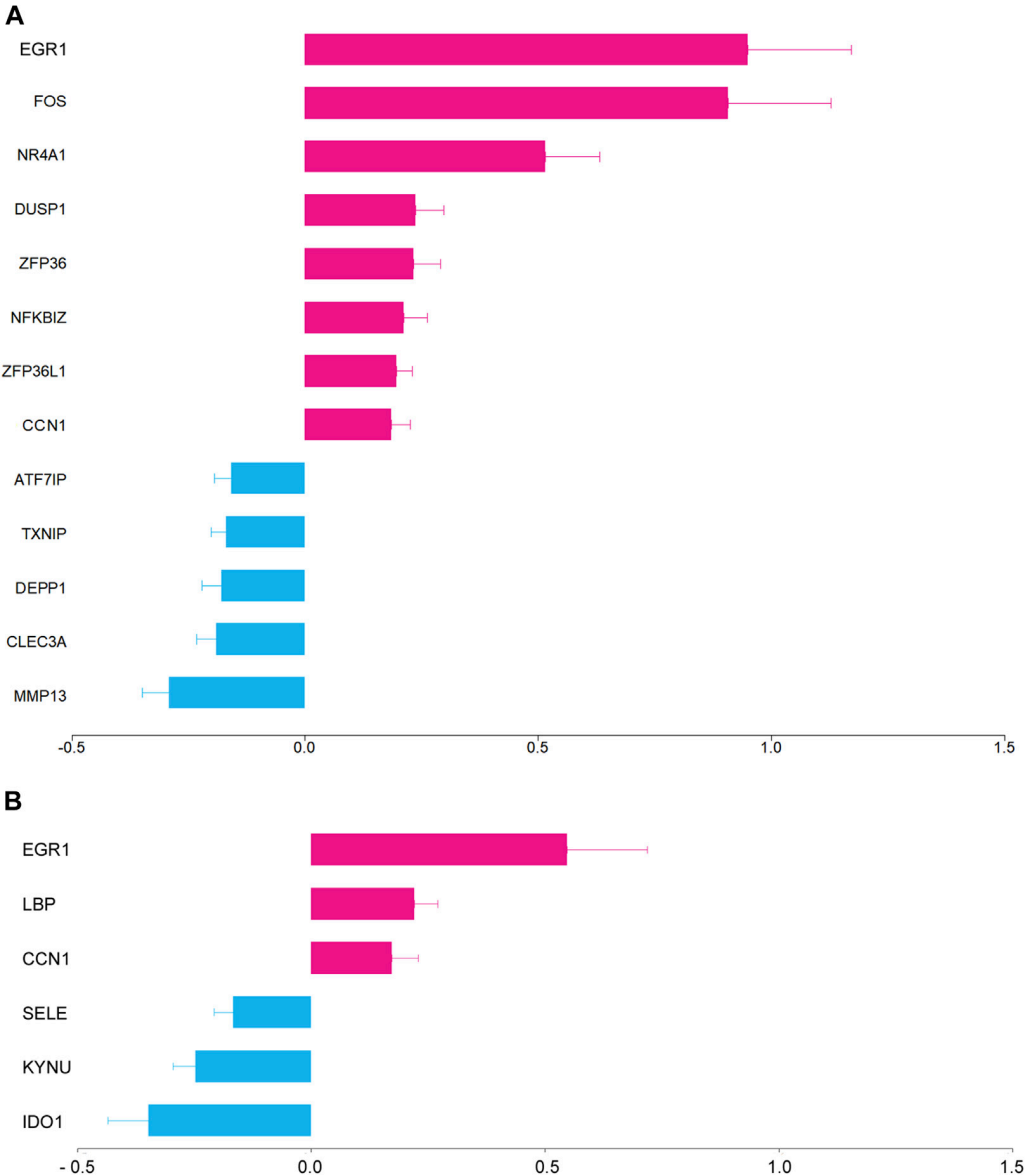
Differentially expressed genes by Ze14 20% (v/v) with Fold Change >1.1 or <0.9, expressed as log2 Fold Change (mean ± SE, *n* = 10). **(A)**: without IL-1β treatment, **(B)**: with IL-1β 10^–11^ M treatment.

According to the DESeq2 analysis and in basal condition, Ze14 20% (v/v) significantly modified the expression of 13 genes by at least ± 10% of Fold Change with a padj <0.05 (Table 1; Figure 1A, full results in Supplementary File 6). Ze14 10% (v/v) failed to significantly modify gene expression.

**TABLE 1:** Differentially expressed genes in chondrocytes treated with Ze14 20% (v/v) compared to saline control using the DESeq2 R package designed for RNA-seq differential gene expression paired-analysis. baseMean is the mean of normalized counts of all samples, normalizing for sequencing depth. padj: False Discovery Rate (FDR) adjusted *p*-value (for details see material and method, statistics paragraph).

	GeneName	baseMean	log2 Fold Change	Fold Change	*p*‐value	padj
*ATF7IP*	Activating transcription factor 7 interacting protein	1249	−0.1585	0.896	2.01E-05	0.0153
*DEPP1*	DEPP1, autophagy regulator	1649	−0.1792	0.883	2.71E-05	0.0189
*CLEC3A*	C-type lectin domain family 3 member A	1132	−0.1902	0.877	7.14E-06	0.0108
*CCN1*	Cellular communication network factor 1	26,310	0.1845	1.136	4.81E-06	0.0087
*DUSP1*	Dual specificity phosphatase 1	738	0.2363	1.178	8.38E-05	0.0448
*EGR1*	Early growth response 1	1468	0.9481	1.929	1.93E-05	0.0153
*FOS*	Fos proto-oncogene, AP-1 transcription factor subunit	188	0.9058	1.874	4.44E-05	0.0269
*MMP13*	Matrix metallopeptidase 13	566	−0.2924	0.817	2.73E-07	0.0008
*NFKBIZ*	NFKB inhibitor zeta	685	0.2116	1.158	1.61E-05	0.0146
*NR4A1*	Nuclear receptor subfamily 4 group a member 1	201	0.5149	1.429	8.76E-06	0.0111
*TXNIP*	Thioredoxin interacting protein	2047	−0.1696	0.889	2.29E-07	0.0008
*ZFP36*	ZFP36 ring finger protein	599	0.2327	1.175	4.24E-05	0.0269
*ZFP36L1*	ZFP36 ring finger protein like 1	4000	0.1950	1.145	1.67E-08	0.0002

The treatment dose-response of each patient sample is illustrated in the Supplementary File 7A (upregulated DEGs) and 7B (downregulated DEGs).

### IL-1β Induced the Expression of Proinflammatory Genes in Human OA Chondrocytes

IL-1β treatment induced a strong upregulation of proinflammatory cytokines including chemokines. Top 12 cytokines produced by OA chondrocytes were in descending order of expression: CXCL8 (∼20.000-fold increase), IL-6, CXCL1, CCL20, CXCL6, CCL2, CXCL3, CXCL2, CXCL5, CCL5, IL-11, and CCL8. CXCL12 and CXCL14 were downregulated by IL-1β. Complete results of differentially expressed genes (DEGs) with IL-1β treatment are in Supplementary File 8. In presence of IL-1β 10^–11^ M, Ze14 20% (v/v) modified the expression of lipopolysaccharide binding protein (LBP, +17%, padj = 0.0126) and E-selectin (SELE, −11%, padj = 0.0252) (Figure 1B). Furthermore, Ze14 also increased the expression of CCN1 (+12%, *p* = 0.0026) and EGR1 (+46%, *p* = 0.0014) and reduced the expression of two genes encoding key enzymes of the kynurenine pathway of tryptophan degradation, kynureninase (KYNU, −16%, *p* = 2.84*10^–7^) and indoleamine 2,3-dioxygenase 1 (IDO1, −21% *p* = 5.96*10^–5^) that had been upregulated by IL-1β (5.78 and 8.81 log2 Fold Change, respectively). Complete results are in the Supplementary Files 7C and 9.

### Ze14 Promoted Extracellular Matrix Formation Through CCN1 Production

**FIGURE 2:**
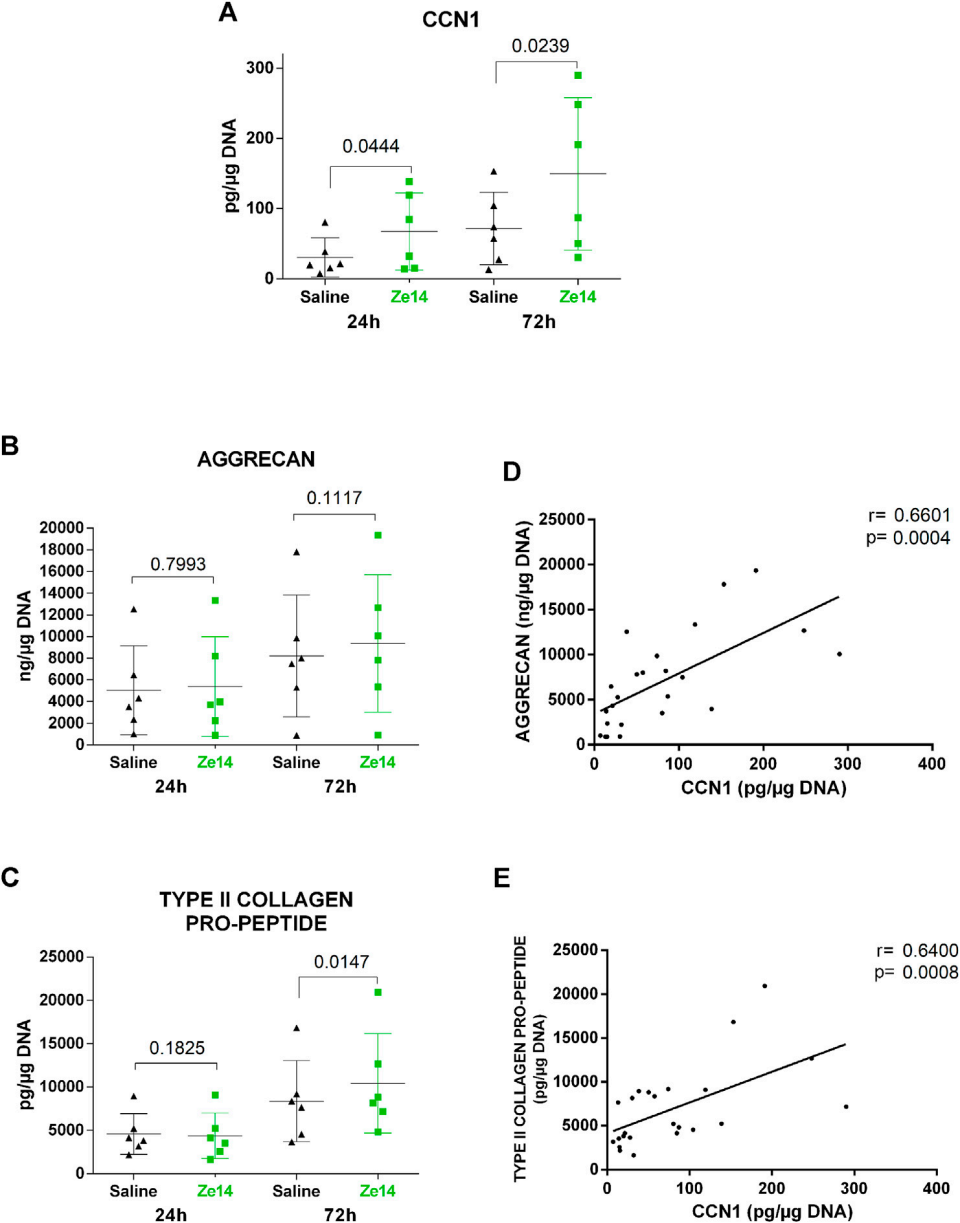
Effect of Ze14 on CCN1, aggrecan and type II collagen pro-peptide production by OA chondrocytes cultured as monolayer (*n* = 6). Saline-treated cells served as controls. Results are shown as mean ± SD of the cultures (*n* = 6). Each dot represents individual culture means. Data were statistically analyzed using paired *t*-test, correlations were analyzed using Pearson’s for aggrecan and Spearman’s for type II collagen pro-peptide.

Cellular communication network factor 1 (CCN1), also known as CYR61, is a growth factor-inducible immediate-early gene, induced notably by TGF-β, shown to be important in chondrogenesis (Chijiiwa et al., 2015). The stimulating effect of Ze14 20% (v/v) on CCN1 production was confirmed by immunoassay. CCN1 was assayed in the culture supernatant of six monolayer chondrocyte cultures from six independent OA patients. Ze14 significantly increased (2.3-fold ± 1.2 after 24 h, *p* = 0.0444 and 2.3-fold ± 1.0 after 72 h, *p* = 0.0239) the CCN1 protein production by human OA chondrocytes (Figure 2A). Because CCN1 inhibits the activity of ADAMTS-4, an important enzyme involved in aggrecan degradation, and has been shown to increase aggrecan and type II collagen synthesis in chondrocytes (Wong et al., 1997; Chijiiwa et al., 2015), we also quantified the protein production of both molecules in these cell cultures. After 72 h, Ze14 20% (v/v) slightly but not significantly increased aggrecan production (14 ± 19%, *p* = 0.1117, Figure 2B) and significantly increased type II collagen pro-peptide production by 27 ± 20% (*p* = 0.0147, Figure 2C). For both time points, CCN1 production by OA chondrocytes was positively and significantly correlated with aggrecan (r = 0.66, *p* = 0.0004, Figure 2D) and type II collagen pro-peptide (r = 0.64, *p* = 0.0008, Figure 2E) production.

### Ze14 Decreased the Production of Pro-MMP-13 in a Hypertrophy-Independent Pathway

**FIGURE 3:**
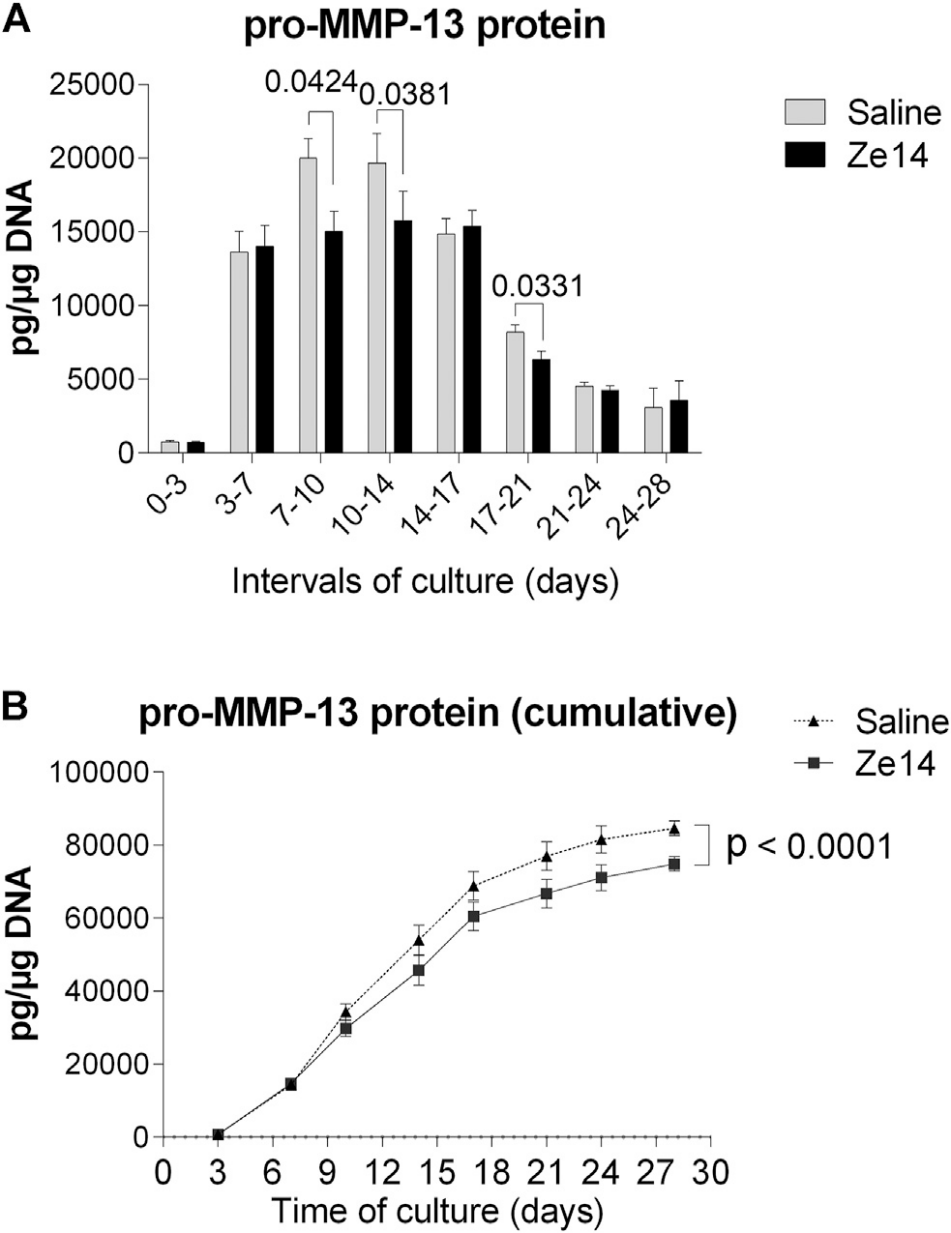
Effects of Ze14 20% (v/v) on pro-MMP-13 production by chondrocytes in alginate beads culture. Saline-treated cells served as controls. Results are shown as mean ± SD of the cultures (*n* = 4). Data were statistically analyzed using paired one-way ANOVA **(A)** or two-way ANOVA **(B)**.

RNA-seq revealed that Ze14 acts on matrix metallopeptidase 13 as well as on several independent genes involved in MMP13 regulation and activation (Figure 3A). MMP-13 is a major enzyme involved in OA pathology: it is both a marker for hypertrophy in chondrocytes and is known to function as an extracellular matrix-degrading enzyme in OA joints (Li et al., 2017). To investigate MMP-13 production during hypertrophic chondrocyte differentiation, we cultured primary OA chondrocytes for 28 days in the alginate beads model in the presence of 10% FBS. We have previously shown that articular chondrocytes become hypertrophic between 21 and 28 days of culture in these conditions (Pesesse et al., 2013; Pesesse et al., 2014). Fresh Ze14 20% (v/v) was added to the culture media twice a week during these 28 days.

**FIGURE 4:**
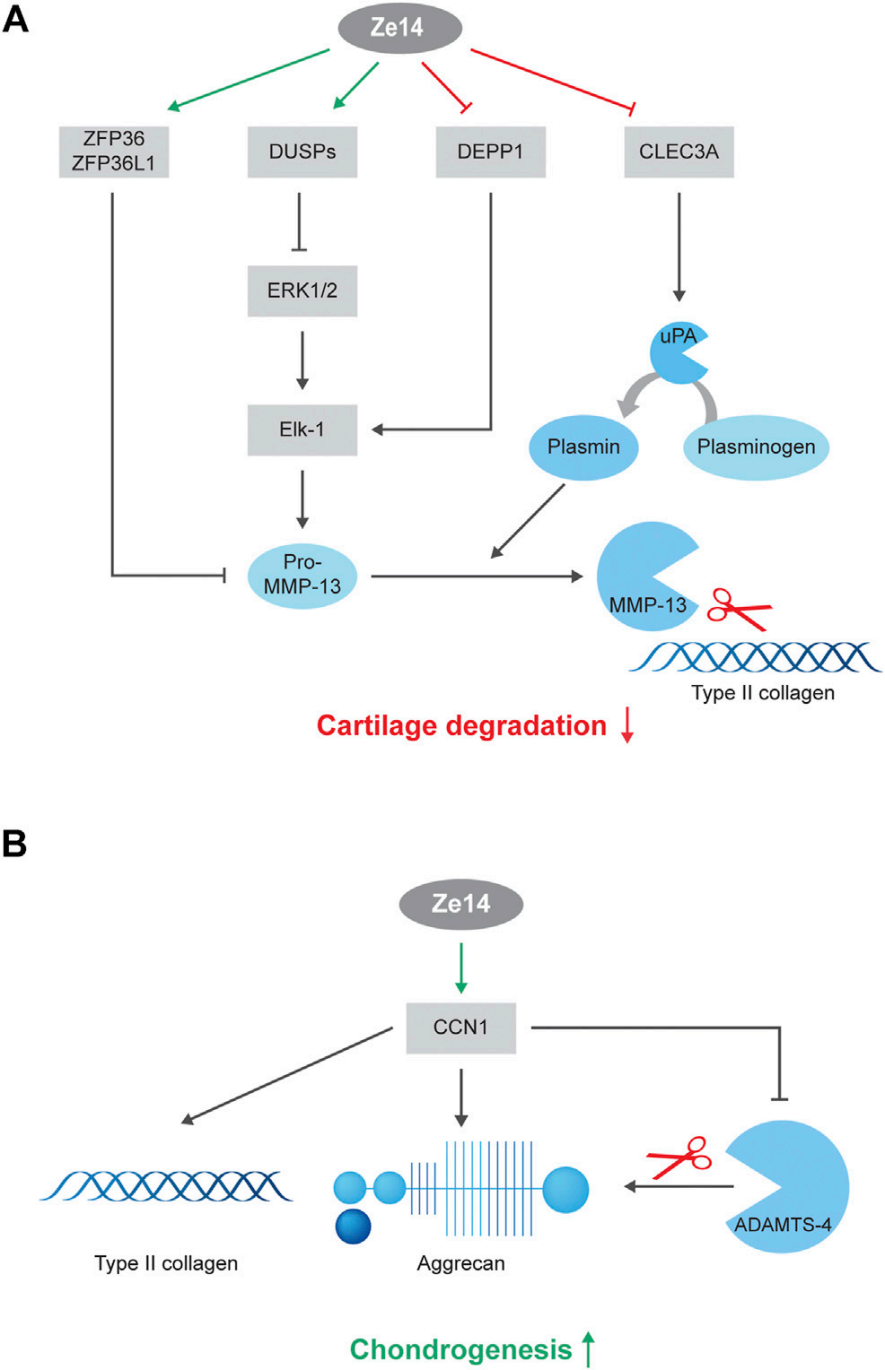
**(A)**: Mechanisms of action of Ze14 on MMP-13 production by chondrocytes: Ze14 significantly modified the expression of DUSP1, DEPP1, ZFP36, ZFP36L1 and CLEC3A, which are pathway mediators involved in MMP13 expression and activation, and reduced the production of pro-MMP-13. This may limit cartilage degradation. Upregulation and downregulation by Ze14 are indicated in green and red color, respectively. **(B)**: Mechanisms of action of Ze14 on chondrogenesis: Ze14 has a proanabolic effect on chondrocytes arguably through CCN1 production. Ze14 increased type II collagen and CCN1 production. Green color indicate upregulation by Ze14.

In the alginate beads model, pro-MMP-13 production highly increased between day 3 and day 14 and then decreased independent of the treatment (Figure 4A). Interestingly, pro-MMP-13 was significantly decreased in Ze14-treated cultures from day 7–14 (from −16 to −25%, *p* < 0.05) and from day 17–21 (−22%, *p* = 0.0331) in comparison to saline-treated control cultures. The cumulative pro-MMP-13 production over 28 days was significantly lower in Ze14-treated chondrocyte cultures than in saline-treated control cultures (*p* < 0.0001, Figure 4B).

To analyze the Ze14 effect on the terminal hypertrophy differentiation, we investigated AP activity and type X collagen gene expression. These markers of hypertrophy increased with culture time, confirming that an induction of hypertrophy took place in these experimental conditions (data not shown). Ze14 did not modify AP activity or COL10A1 expression (Supplementary File 10).

## Discussion

In this study, we aimed to identify molecular pathways in human OA chondrocytes treated with Ze14. We used the latest NGS technology to generate the transcriptome, capturing dynamic changes in chondrocytes induced by the treatment, compared to saline control. We extended our transcript-level analysis to the protein level by directly measuring proteins with ELISA. Here we demonstrate that Ze14 promotes extracellular matrix formation arguably through CCN1 production and reduced pro-MMP-13, which may limit cartilage degradation. These findings may explain some effects observed in the clinical studies with OA patients.

Most of Ze14 DEGs were involved in MMP-13, also known as collagenase 3, regulation pathways (Figure 4A), including expression of MMP13 itself. It is known that MMP13 is significantly overexpressed in the joints and articular cartilage in patients with OA, and is a major proteinase involved in the degradation of type II collagen, a key constituent of the extracellular matrix (Li et al., 2017). Indeed, Ze14 modified DUSP1, DEPP1, ZFP36, ZFP36L1, and CLEC3A, which are signaling factors regulating MMP13 expression and activation. Furthermore, long-term (28 days) treatment with Ze14 significantly reduced the production of pro-MMP-13, the inactive precursor of MMP-13. The data analysis suggests that Ze14 potentially limits type II collagen degradation by reducing MMP-13 production and could have a beneficial effect on cartilage degradation.

Other notable results of this study are the stimulating effects of Ze14 on type II collagen and CCN1 production in human OA chondrocytes showing that Ze14 has proanabolic properties on cartilage.

During chondrogenic differentiation, chondrocytes express a mature chondrogenic phenotype (a set of characteristics specific to mature chondrocytes present in healthy cartilage). In adult cartilage, it involves the synthesis of matrix components, mainly type II collagen and aggrecan, two molecules characteristic of cartilage extracellular matrix, without changing the cell number. CCN1 is a growth factor-inducible immediate-early gene, directly involved in chondrogenesis: in micromass culture, purified recombinant CCN1 protein promoted chondrogenesis demonstrated by the expression of type II collagen, increased [35 S] sulfate incorporation, and larger alcian-blue staining of cartilage nodules used to assess aggrecan. Aggrecan is the major proteoglycan of the cartilage matrix. (Wong et al., 1997; Chijiiwa et al., 2015). Furthermore, CCN1 inhibits the activity of aggrecanase-1, also known as ADAMTS-4, an important enzyme involved in aggrecan degradation. Arguably, by increasing CCN1, Ze14 could prevent aggrecan degradation and increase the synthesis of type II collagen and aggrecan (proanabolic effect). Interestingly, we found that type II collagen and aggrecan production were correlated with CCN1 production, suggesting that CCN1 could be a signaling factor involved in Ze14’s anabolic effect on chondrocytes. The correlation between aggrecan and CCN1 has previously also been observed using alcian-blue staining (Wong et al., 1997).

These results demonstrate that Ze14 increases cartilage matrix formation in OA chondrocytes, arguably via CCN1, promoting chondrogenesis (Figure 4B). That is an important finding since chondrocytes lose their “healthy” chondrogenic properties and change into hypertrophic, catabolic, or fibroblastic chondrocytes as OA develops and progresses. However, in our *in vitro* hypertrophy model, Ze14 failed to stop or delay the terminal hypertrophic differentiation of chondrocytes. One reason could be that activation of hypertrophic differentiation pathways in chondrocytes obtained from our patients might have already been too advanced. Using healthy chondrocytes from young donors could reveal different outcomes. Furthermore*,* additional studies are needed to investigate the effect of Ze14 on fibroblastic chondrocytes.

Interestingly, Ze14 increased the expression of two key signaling factors involved in chondrogenesis: Early Growth Response (EGR)1 and FOS. SOX9 is upregulated through the induction of EGR1, EGR3 and FOS mRNA (Spaapen et al., 2013). SOX9 is the transcription factor involved in chondrogenesis known to increase the expression of type II collagen and aggrecan in chondrocytes (Oh et al., 2010). Arguably, the upregulation of EGR1 and FOS by Ze14 has a beneficial effect on chondrogenesis and potentially leads to an increased anabolism.

Finally, we have observed that Ze14 may modulate the harmful effect of IL-1β on chondrocyte metabolism. IL-1β is considered as one of the major proinflammatory mediators in OA. Besides, IL-1β is a potent stimulatory and deleterious cytokine, and IL-1β stimulation of chondrocytes is the most widely used *in vitro* model in OA. In the presence of IL-1β 10^–11^ M, Ze14 20% (v/v) increased CCN1, LBP, and EGR1, and decreased expression of E-selectin and of two enzymes of the kynurenine pathway of tryptophan degradation, kynureninase and indoleamine 2,3-dioxygenase 1.

E-selectin is an adhesion molecule mediating the initial rolling of leukocytes along the surface of the vascular endothelium before firm adhesion and migration of the leukocytes occurs. It has been reported that both P- and E-selectin are expressed on the vascular endothelium of the synovium in rheumatoid arthritis (RA), and high levels of soluble E-selectin are detectable in the synovial fluid in RA. E-selectin plays an important role early in the development of adjuvant-induced arthritis in the rat (Issekutz et al., 2001). In our study, E-selectin was highly upregulated with IL-1β (12.6 log2 Fold Change) and was reduced by Ze14 treatment. Therefore, we assume that the initial rolling and the subsequent recruitment of leukocytes is inhibited in the presence of Ze14, leading to a reduction of inflammation. To confirm the potential effect on inflammation, further investigations would be necessary.

Another important effect of Ze14 during IL-1β-treatment was observed on two enzymes of the kynurenine pathway of tryptophan degradation, kynureninase and indoleamine 2,3-dioxygenase 1. These enzymes were highly upregulated with IL-1β stimulation (5.78 and 8.81 log2 Fold Change, respectively), and were decreased after Ze14 treatment (−16% and −21%). Elevated tryptophan metabolism and kynurenine levels have also been shown in primary synovial cell cultures in response to elevated interferon-γ, suggesting altered or increased tryptophan metabolism in response to inflammatory cytokines associated with arthritis (Malone et al., 1994). In this instance, further understanding of the impact of disease progression on tryptophan/kynurenine metabolism could benefit from further analysis of this pathway in OA.

In conclusion, in primary OA chondrocyte cultures, Ze14 promoted extracellular matrix formation probably through CCN1 production, a growth factor well-correlated with type II collagen and aggrecan production. Ze14 also significantly modified the expression of DUSP1, DEPP1, ZFP36, ZFP36L1, and CLEC3A, which are pathway mediators involved in MMP13 expression and activation. Long-term treatment with Ze14 significantly decreased pro-MMP-13 production, which is the inactive precursor of the metallopeptidase 13 involved in type II collagen degradation. For confirmation whether the effect of Ze14 on pro-MMP-13 has indeed an impact on type II collagen and cartilage matrix degradation, further investigations would be necessary, such as the analysis of protein activation (pro-MMP-13 to active MMP-13), the determination of MMP-13 enzymatic activity, and the analysis of type II collagen degradation biomarkers *in vivo*.

Our analyses show that Ze14 is a multitarget medication reducing several characteristics of OA: it decreased pro-MMP-13, potentially inhibiting catabolism, and demonstrated anabolic properties probably driven by CCN1, leading to a global net increase of chondrogenesis, characterized by a stimulation of type II collagen synthesis.

## Data Availability

The datasets presented in this study can be found in online repositories. The names of the repository/repositories and accession number(s) can be found below: Gene Expression Omnibus (GEO) repository, under accession number GSE162510 (https://www.ncbi.nlm.nih.gov/geo/query/acc.cgi?&acc=GSE162510).

## Abbreviations

ADAMTS-4, a disintegrin and metalloproteinase with thrombospondin motifs 4; AP, alkaline phosphatase; CM, cells with associated matrix; CYR61, cysteine-rich angiogenic inducer 61; DMEM, Dulbecco’s Modified Eagle’s Medium; ECM, extracellular matrix; EGR, early growth response; FBS, fetal bovine serum; FDR, false discovery rate; HEPES, 4-(2-hydroxyethyl)-1-piperazineethanesulfonic acid; IL, interleukin; LDH, lactate dehydrogenase; MMP, matrix metallopeptidase; NGS, next-generation sequencing; OA, osteoarthritis; padj, p-value adjusted; RA, rheumatoid arthritis; RT-PCR, reverse transcription-polymerase chain reaction; RIN, RNA integrity number; RNA, ribonucleic acid; ROS, reactive oxygen species; SD, standard deviation; SE, standard error; TKR, total knee replacement; TNF, tumor necrosis factor.
