# Supplementary material for: Reduction of Matrix Metallopeptidase 13 and Promotion of Chondrogenesis by Zeel T in Primary Human Osteoarthritic Chondrocytes
Source: Front Pharmacol. 2021 May 11;12:635034. doi: 10.3389/fphar.2021.635034 (PMC8144641; doi:10.3389/fphar.2021.635034)
Supplement: Supplementary file 1 [file DataSheet1.zip › Supplementary files/635304_Supplementary File 1.pptx]

## Slide 1
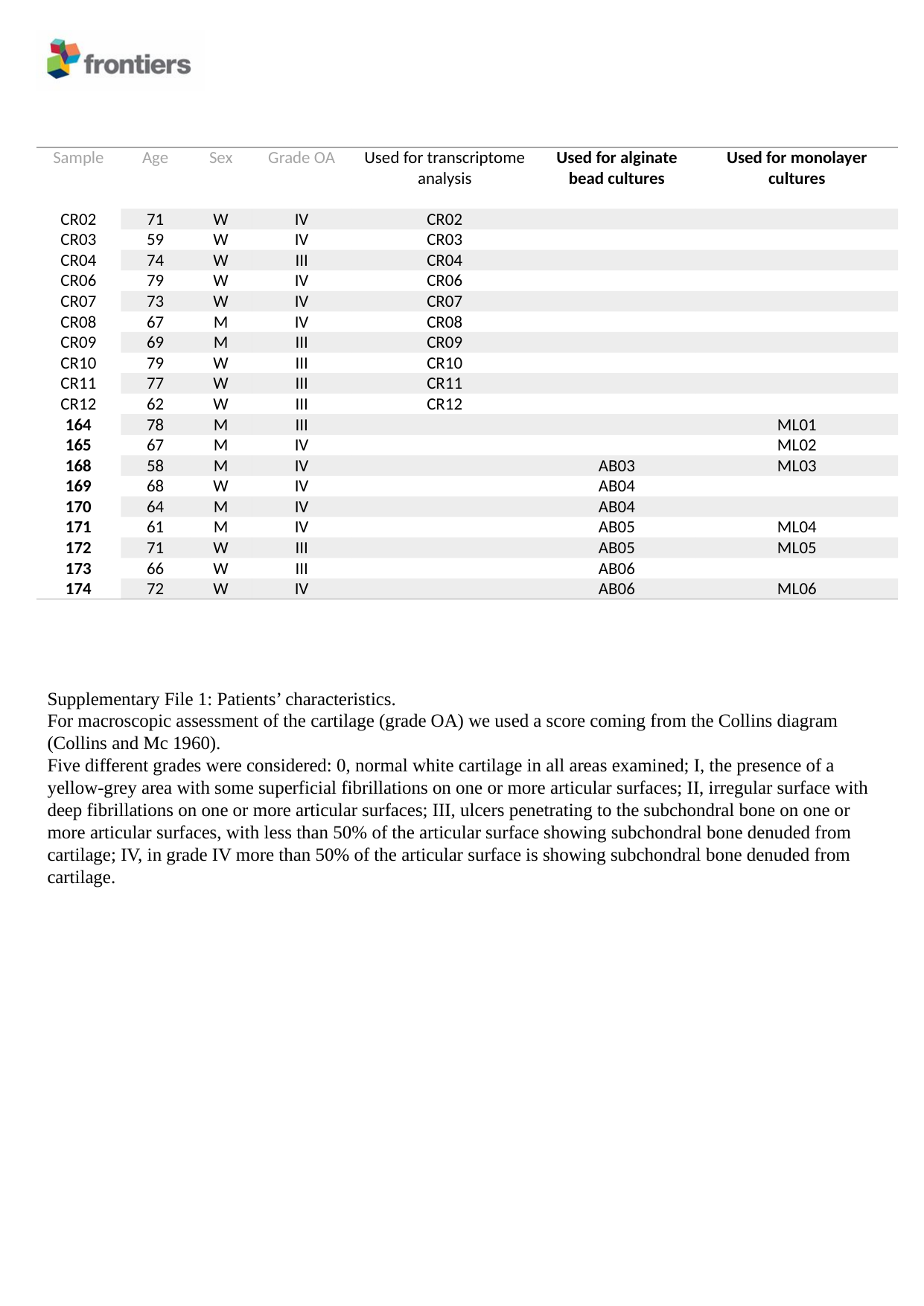

| Sample | Age | Sex | Grade OA | Used for transcriptome analysis | Used for alginate bead cultures | Used for monolayer cultures |
| --- | --- | --- | --- | --- | --- | --- |
| CR02 | 71 | W | IV | CR02 | | |
| CR03 | 59 | W | IV | CR03 | | |
| CR04 | 74 | W | III | CR04 | | |
| CR06 | 79 | W | IV | CR06 | | |
| CR07 | 73 | W | IV | CR07 | | |
| CR08 | 67 | M | IV | CR08 | | |
| CR09 | 69 | M | III | CR09 | | |
| CR10 | 79 | W | III | CR10 | | |
| CR11 | 77 | W | III | CR11 | | |
| CR12 | 62 | W | III | CR12 | | |
| 164 | 78 | M | III | | | ML01 |
| 165 | 67 | M | IV | | | ML02 |
| 168 | 58 | M | IV | | AB03 | ML03 |
| 169 | 68 | W | IV | | AB04 | |
| 170 | 64 | M | IV | | AB04 | |
| 171 | 61 | M | IV | | AB05 | ML04 |
| 172 | 71 | W | III | | AB05 | ML05 |
| 173 | 66 | W | III | | AB06 | |
| 174 | 72 | W | IV | | AB06 | ML06 |
Supplementary File 1: Patients’ characteristics.
For macroscopic assessment of the cartilage (grade OA) we used a score coming from the Collins diagram (Collins and Mc 1960).
Five different grades were considered: 0, normal white cartilage in all areas examined; I, the presence of a yellow-grey area with some superficial fibrillations on one or more articular surfaces; II, irregular surface with deep fibrillations on one or more articular surfaces; III, ulcers penetrating to the subchondral bone on one or more articular surfaces, with less than 50% of the articular surface showing subchondral bone denuded from cartilage; IV, in grade IV more than 50% of the articular surface is showing subchondral bone denuded from cartilage.
