# Supplementary material for: Reduction of Matrix Metallopeptidase 13 and Promotion of Chondrogenesis by Zeel T in Primary Human Osteoarthritic Chondrocytes
Source: Front Pharmacol. 2021 May 11;12:635034. doi: 10.3389/fphar.2021.635034 (PMC8144641; doi:10.3389/fphar.2021.635034)
Supplement: Supplementary file 1 [file DataSheet1.zip › Supplementary files/635304_Supplementary File 2.docx]

**Supplementary File 2: Composition of Ze14 injection solution**

| **Component** | | **Manufacturing method (Ph. Eur.)** | **µg/mL** |
| --- | --- | --- | --- |
| *Plant extracts* | | | |
|  | Extract from dried underground parts of *Arnica montana* L. (Arnica) | Method 1.1.8 | 100 |
|  | Extract from fresh young twigs of *Rhus toxicodendron* L. (*Toxicodendron quercifolium*MICHX. GREENE) which are not yet lignified. (Poison ivy) | Method 1.1.3 | 100 |
|  | Extract from dried underground parts of Sanguinaria canadensis L. (Bloodroot) | Method 1.1.8 | 1.5 |
|  | Extract from fresh shoots of *Solanum dulcamara*L. collected prior to flowering (Bittersweet nightshade) | Method 1.1.3 | 10 |
|  | Extract from fresh underground parts of *Symphytum officinale* L. collected prior to flowering (Comfrey) | Method 1.1.5 | 0.015 |
| *Chemical substances* | | | |
|  | Coenzyme A | Method 3.1.1 | 1E-5 |
|  | Nicotinamide adenine dinucleotide | Method 3.1.1 | 1E-5 |
|  | Sodium diethyl oxalacetate | Method 4.1.1 | 1E-5 |
|  | Sulfur | Method 4.1.1 | 0.002 |
|  | Thioctic acid | Method 4.1.1 | 1E-5 |
| *Extracts of organs from healthy pigs* | | | |
|  | Cartilage | Method 2.1.1 | 0.001 |
|  | Fully developed embryo | Method 2.1.1 | 0.001 |
|  | Umbilical cord | Method 2.1.1 | 0.001 |
|  | Placenta | Method 2.1.1 | 0.001 |

Ph. Eur. = European Pharmacopoeia

The medicinal product is prepared according homeopathic manufacturing procedures.
