# Supplementary material for: Reduction of Matrix Metallopeptidase 13 and Promotion of Chondrogenesis by Zeel T in Primary Human Osteoarthritic Chondrocytes
Source: Front Pharmacol. 2021 May 11;12:635034. doi: 10.3389/fphar.2021.635034 (PMC8144641; doi:10.3389/fphar.2021.635034)
Supplement: Supplementary file 1 [file DataSheet1.zip › Supplementary files/635304_Supplementary File 3.pptx]

## Slide 1
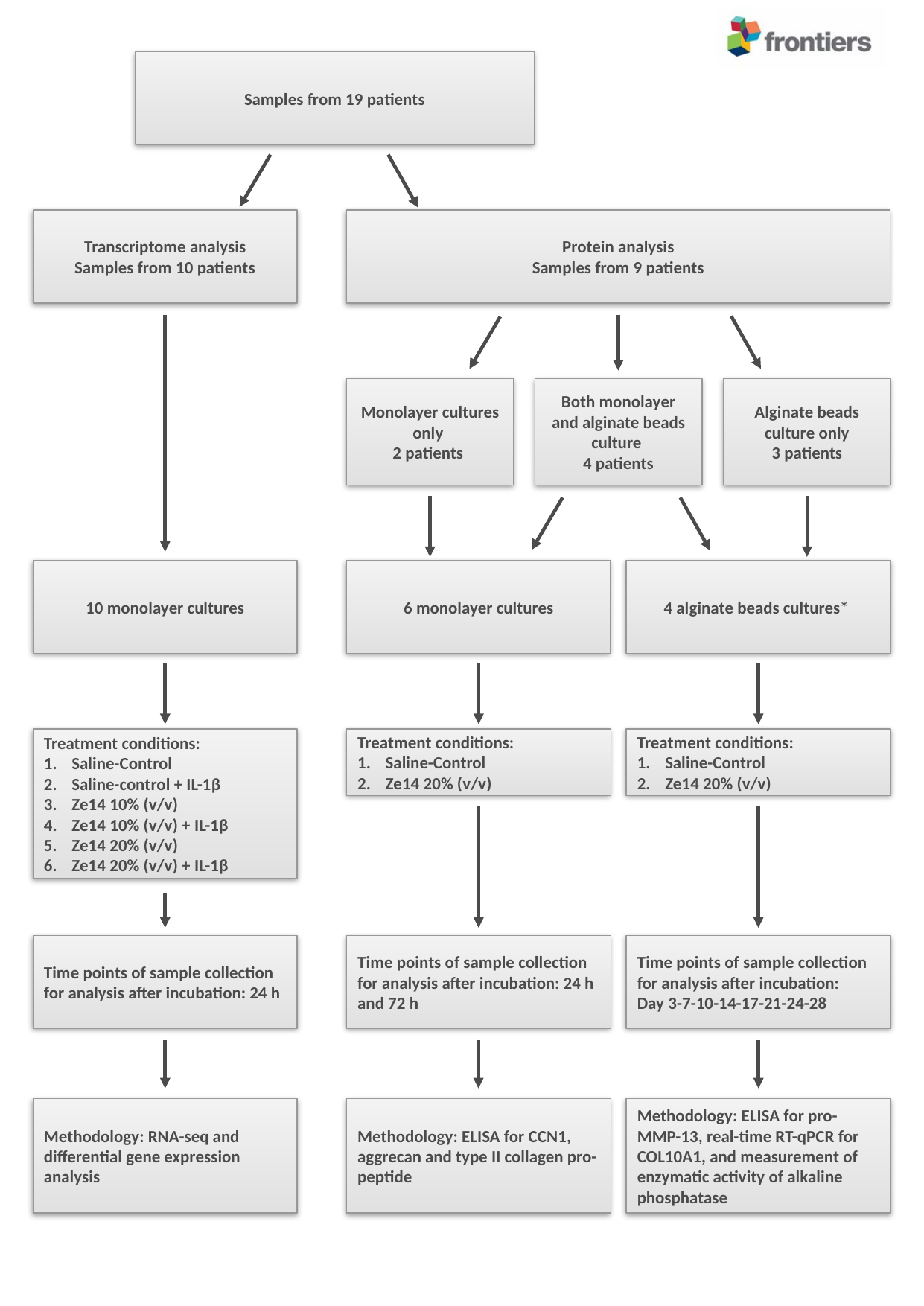

Samples from 19 patients
Transcriptome analysis
Samples from 10 patients
Protein analysis
Samples from 9 patients
Monolayer cultures only
2 patients
Both monolayer and alginate beads culture
4 patients
Alginate beads culture only
3 patients
10 monolayer cultures
6 monolayer cultures
4 alginate beads cultures*
Treatment conditions:
Saline-Control
Saline-control + IL-1β
Ze14 10% (v/v)
Ze14 10% (v/v) + IL-1β
Ze14 20% (v/v)
Ze14 20% (v/v) + IL-1β
Treatment conditions:
Saline-Control
Ze14 20% (v/v)
Treatment conditions:
Saline-Control
Ze14 20% (v/v)
Time points of sample collection for analysis after incubation: 24 h
Time points of sample collection for analysis after incubation: 24 h and 72 h
Time points of sample collection for analysis after incubation:
Day 3-7-10-14-17-21-24-28
Methodology: RNA-seq and differential gene expression analysis
Methodology: ELISA for CCN1, aggrecan and type II collagen pro-peptide
Methodology: ELISA for pro-MMP-13, real-time RT-qPCR for COL10A1, and measurement of enzymatic activity of alkaline phosphatase

## Slide 2
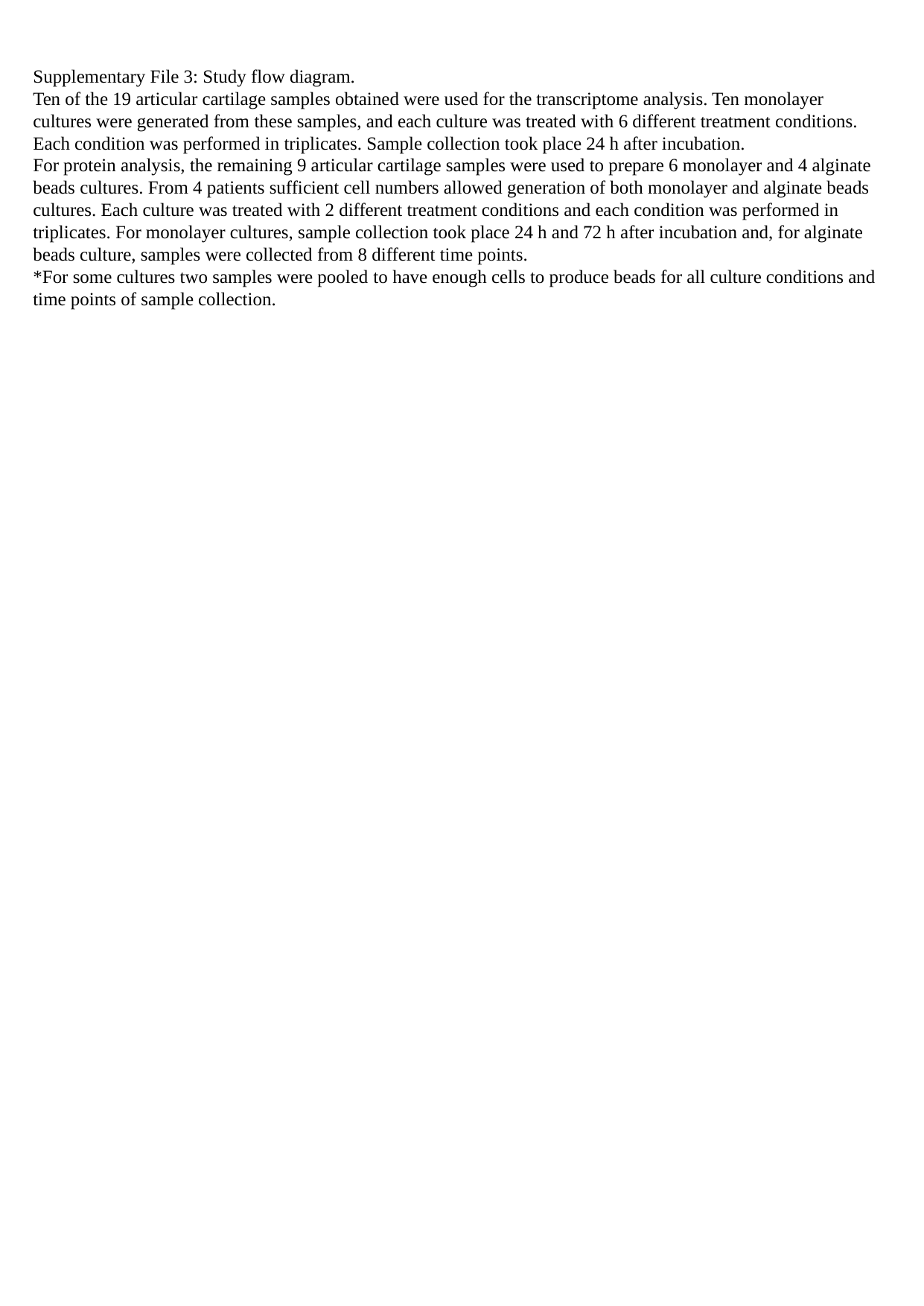

Supplementary File 3: Study flow diagram.
Ten of the 19 articular cartilage samples obtained were used for the transcriptome analysis. Ten monolayer cultures were generated from these samples, and each culture was treated with 6 different treatment conditions. Each condition was performed in triplicates. Sample collection took place 24 h after incubation.
For protein analysis, the remaining 9 articular cartilage samples were used to prepare 6 monolayer and 4 alginate beads cultures. From 4 patients sufficient cell numbers allowed generation of both monolayer and alginate beads cultures. Each culture was treated with 2 different treatment conditions and each condition was performed in triplicates. For monolayer cultures, sample collection took place 24 h and 72 h after incubation and, for alginate beads culture, samples were collected from 8 different time points.
*For some cultures two samples were pooled to have enough cells to produce beads for all culture conditions and time points of sample collection.
