# Supplementary material for: Reduction of Matrix Metallopeptidase 13 and Promotion of Chondrogenesis by Zeel T in Primary Human Osteoarthritic Chondrocytes
Source: Front Pharmacol. 2021 May 11;12:635034. doi: 10.3389/fphar.2021.635034 (PMC8144641; doi:10.3389/fphar.2021.635034)
Supplement: Supplementary file 1 [file DataSheet1.zip › Supplementary files/635304_Supplementary File 5.pptx]

## Slide 1
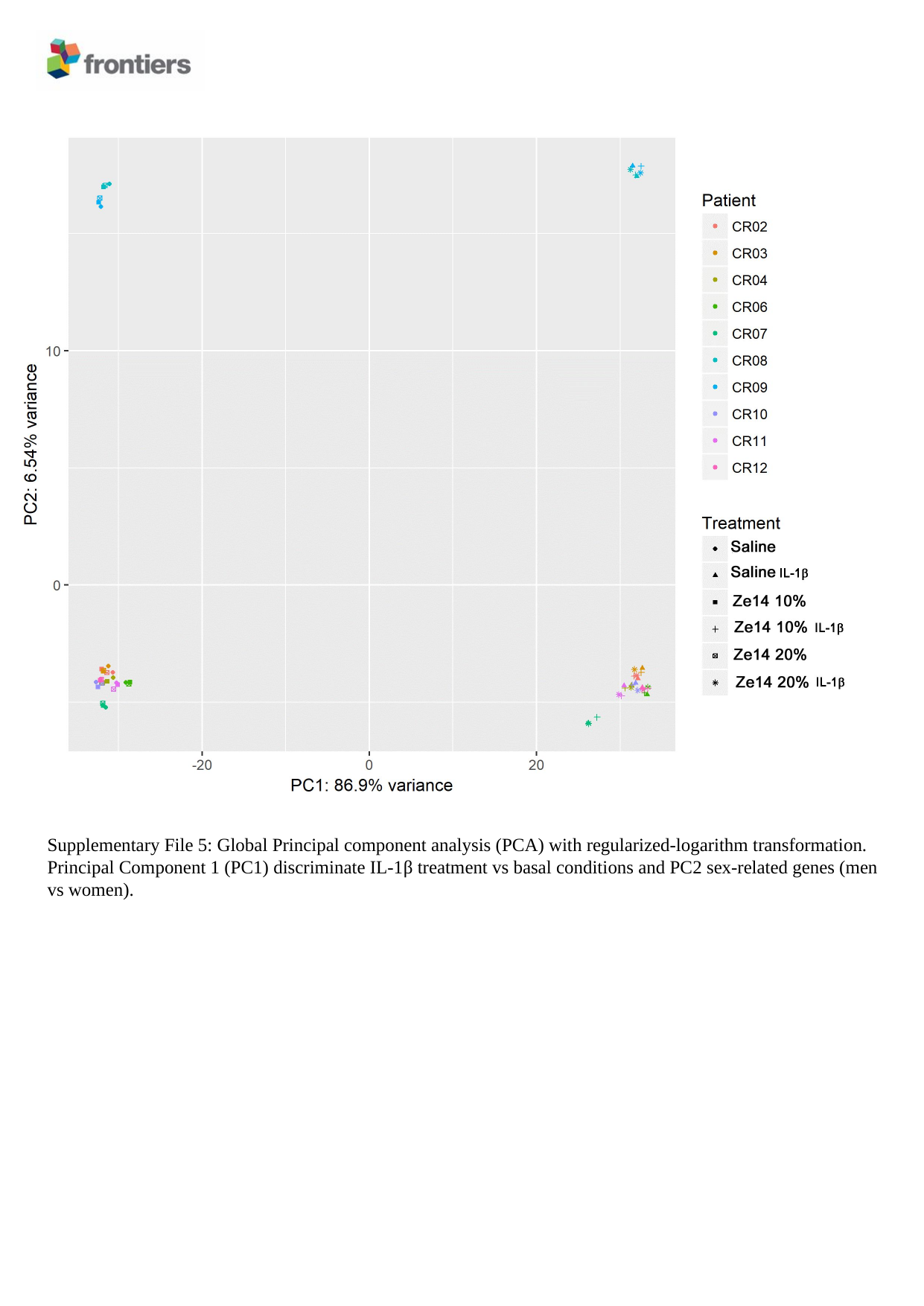

Supplementary File 5: Global Principal component analysis (PCA) with regularized-logarithm transformation.
Principal Component 1 (PC1) discriminate IL-1β treatment vs basal conditions and PC2 sex-related genes (men vs women).
