# Supplementary figures and images for: Reduction of Matrix Metallopeptidase 13 and Promotion of Chondrogenesis by Zeel T in Primary Human Osteoarthritic Chondrocytes
Source: Front Pharmacol. 2021 May 11;12:635034. doi: 10.3389/fphar.2021.635034 (PMC8144641; doi:10.3389/fphar.2021.635034)

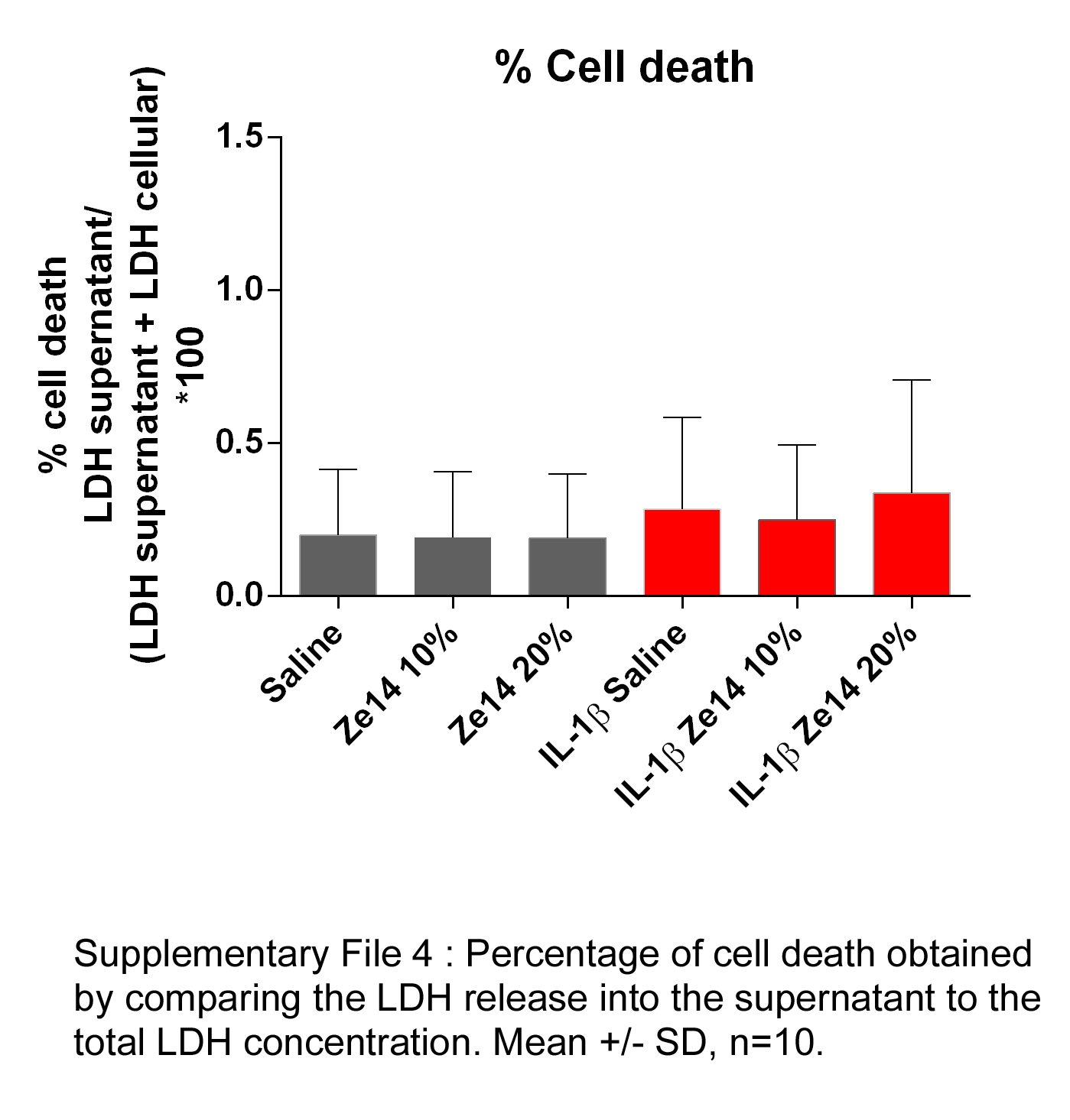

Supplement: Supplementary file 1 [file DataSheet1.zip › Supplementary files/635304_Supplementary File 4.tif]

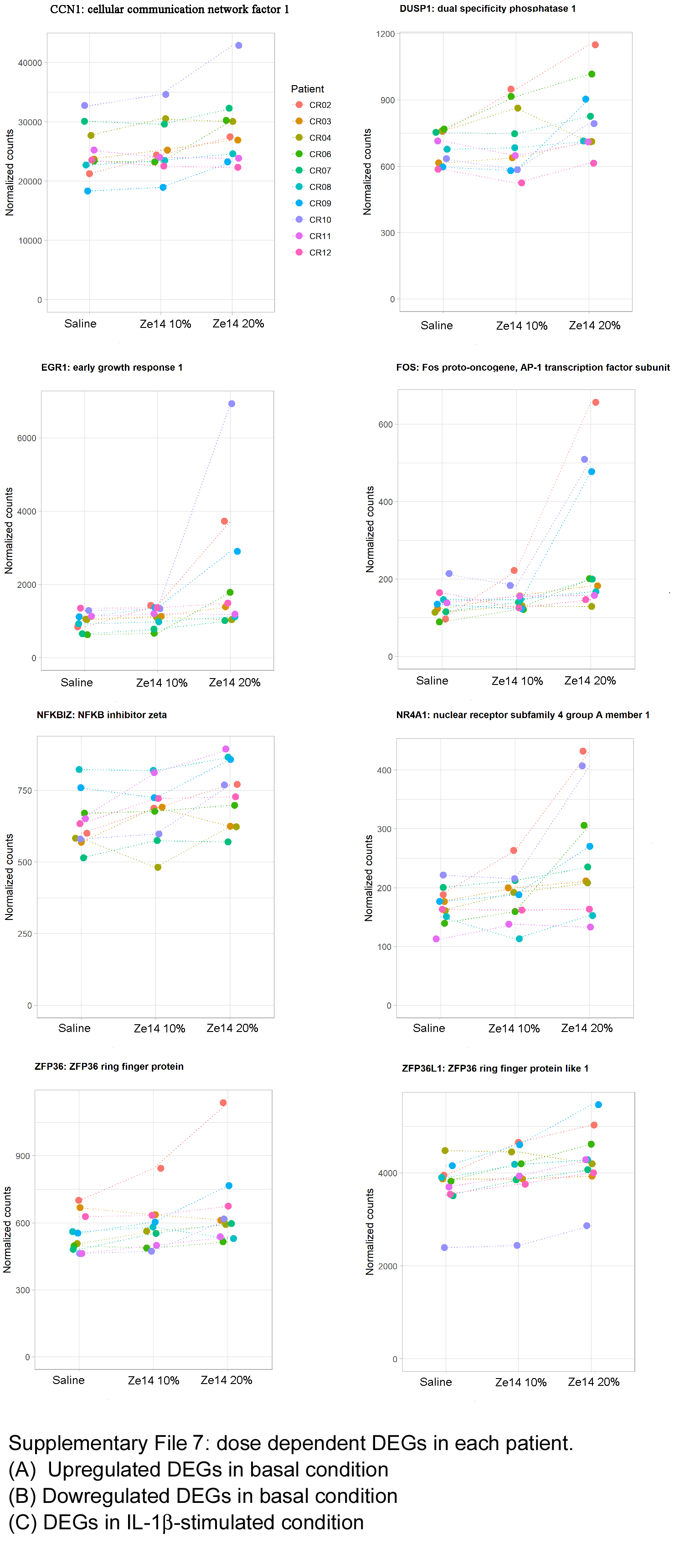

Supplement: Supplementary file 1 [file DataSheet1.zip › Supplementary files/635304_Supplementary File 7A.tif]

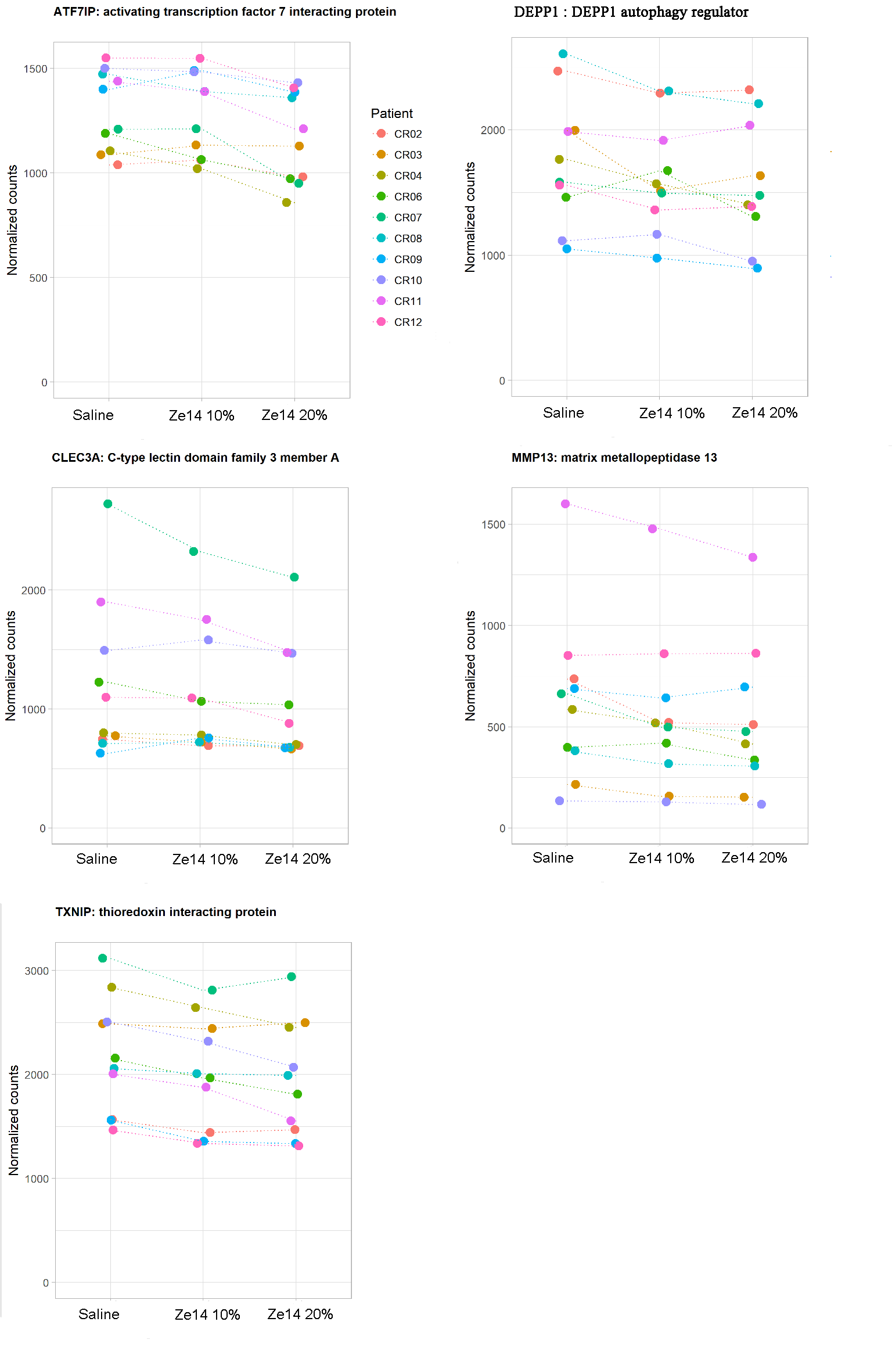

Supplement: Supplementary file 1 [file DataSheet1.zip › Supplementary files/635304_Supplementary File 7B.TIF]

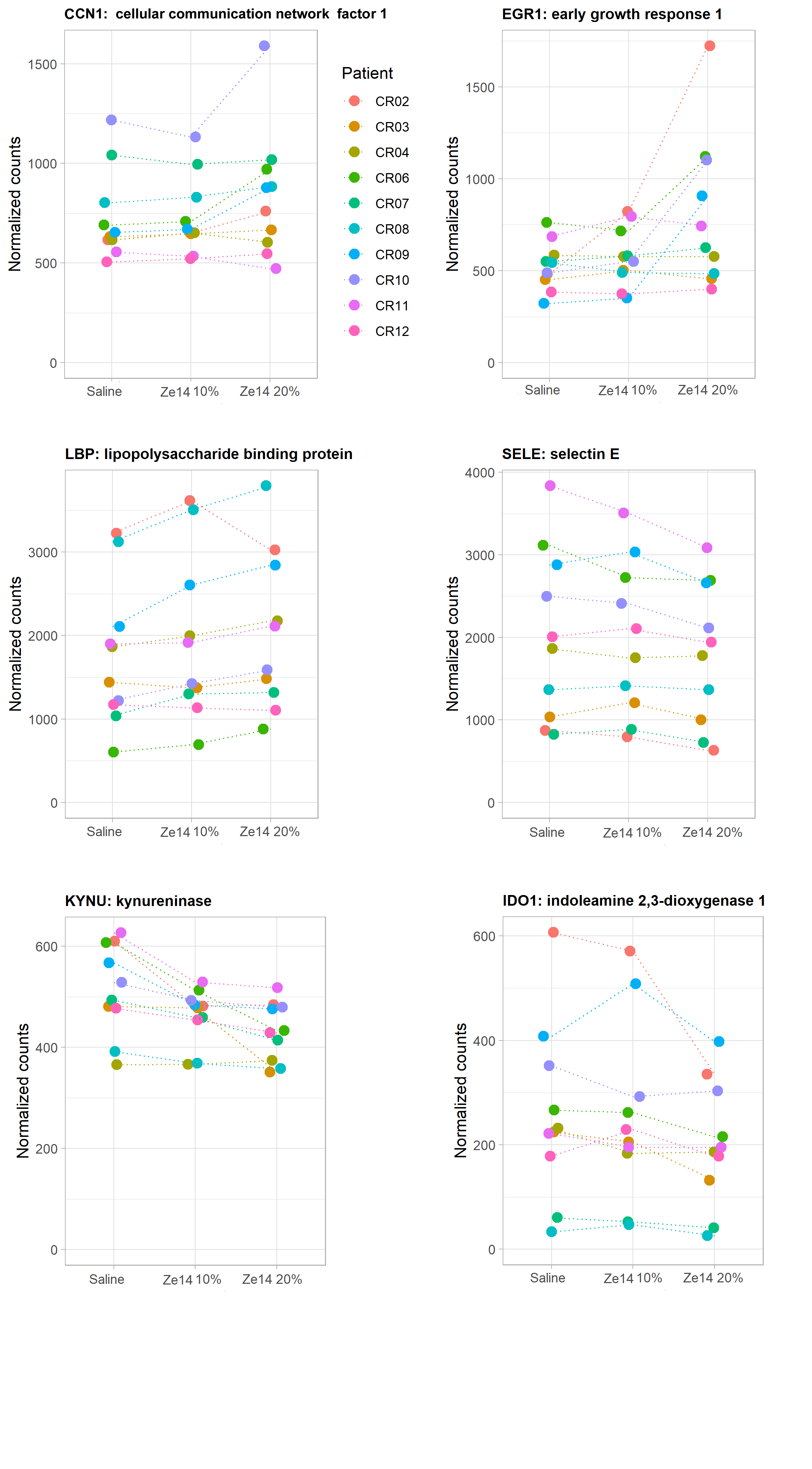

Supplement: Supplementary file 1 [file DataSheet1.zip › Supplementary files/635304_Supplementary file 7C.tif]
